# Population HLA coverage of experimentally characterized *P. falciparum CD8* ^*+*^ T cell epitopes as potential components of a multi-epitope malaria vaccine construct: an *in silico* insight

**DOI:** 10.3389/fbinf.2026.1722563

**Published:** 2026-03-12

**Authors:** Courage Siame, Rafiatu Abdul-Mumin, Georgina Agyekum, Linda Akuffo, Ernest Afari, Cecilia Adwoa Biaa Yankey, Kwadwo Asamoah Kusi

**Affiliations:** Department of Immunology, Noguchi Memorial Institute for Medical Research, College of Health Sciences, University of Ghana, Accra, Ghana

**Keywords:** epitope prediction, HLA, immune epitope database, malaria, *P. falciparum*

## Abstract

**Objective:**

Malaria remains a significant global health challenge, with *Plasmodium falciparum* causing the most severe cases and deaths, particularly in sub-Saharan Africa. An effective universal or pan vaccine is needed to augment current concerted control efforts. This *in silico* study investigated the global population coverage of human leukocyte antigen (HLA) that recognize experimentally validated CD8^+^ T cell epitopes derived from three *P. falciparum* antigens (CSP, AMA1 and TRAP), focusing on their potential for inclusion in a globally effective multi-epitope malaria vaccine.

**Methodology:**

Sixteen experimentally validated CD8^+^ T cell epitopes, together with the HLA typing data of study participants were curated from our previously published work. Using the Immune Epitope Database and Analysis conservancy and population coverage tool, the epitopes were analyzed for their conservancy and ability to bind HLA alleles from fourteen geographic subregions and seventy-eight countries.

**Result:**

CSPep1, CSPep2, CSPep4, CSPep6, CSPep9, CSPep10, CSPep11, and TRAPep1 were 100% conserved across the sixteen strains of *P. falciparum* while the other epitopes had conservancy of 55% or more. The CSP-derived epitope IQNSLSTEW (CSPep5) demonstrated the highest epitope coverage globally (>85%) and in West Africa (93.35%). In contrast, ILSVSSFLF (CSPep4) showed minimal coverage (<5% in Africa, <2% in other populations), and these highlight the impact of HLA diversity on epitope recognition. LLACAGLAYK (TRAPep1) and STEWSPCSV (CSPep10) had >50% population coverage but were highly conserved across diverse *P. falciparum* strains. Spearman’s correlation clustering shows three distinct clusters of epitopes with similar HLA coverage across the subregions.

**Conclusion:**

These findings highlight the importance of integrating host HLA binding promiscuity and parasite epitope conservation in developing universal or pan multi-epitope malaria vaccines. By combining conserved epitopes with broad HLA coverage, this approach promotes wide immune recognition while limiting strain-specific immune evasion. Incorporating relevant HLA class II and antibody epitopes could further enhance immune responses, providing effective protection for both children in malaria-endemic regions and malaria-naïve travelers.

## Introduction

Malaria remains a global health challenge, particularly in the World Health Organization (WHO) Africa region where the disease is endemic. Globally, malaria was responsible for 282 million cases and 610,000 deaths in 2024 (World malaria report 2025). Among the five *Plasmodium* species that infect humans, *P. falciparum* is the most virulent and responsible for the majority of severe malaria cases and deaths, especially in sub-Saharan Africa. The high burden of *Plasmodium falciparum* in Africa is driven by multiple factors, including the presence of efficient mosquito vectors, favorable climatic conditions, and limited access to healthcare resources. Malaria also remains a threat to a significant proportion of the global population and can be especially dangerous for malaria-naïve travelers to disease endemic areas. Despite efforts to control malaria via insecticide-treated bed nets, use of antimalarial drugs, and various vector control measures, the disease continues to pose a significant threat to public health, particularly in resource-limited settings (World malaria report 2025).

The development of an effective malaria vaccine has long been a priority in combating malaria. However, the complex life cycle of *P. falciparum*, in addition to its ability to evade the host immune system, has made development of a highly effective vaccine a challenging endeavor (Hoffman et al., 2015). Contemporary vaccine strategies target different stages of the parasite’s life cycle, including the pre-erythrocytic (sporozoite) stage, the blood stage (merozoite), and the sexual stage (gametocyte) (Tajudeen et al., 2024). Among these, the pre-erythrocytic stage is of strategic focus, as preventing infection at this stage could halt the parasite before it reaches the blood stage to cause clinical disease. The circumsporozoite protein (CSP), an essential antigen that is highly expressed on the surface of sporozoites, has been a key target for vaccine development and is the main vaccine component in the two most advanced malaria vaccines, RTS,S/AS01 and R21/Matrix-M (Datoo et al., 2021; Seidlein, 2025)

In addition to CSP, other antigens such as apical membrane antigen 1 (AMA1), thrombospondin-related anonymous protein (TRAP) and the merozoite surface proteins (MSPs) have also variously been explored as potential vaccine candidates. AMA1 plays a critical role in the invasion of host cells by both sporozoites and merozoites (Cowman et al., 2016; Silvie et al., 2004), and is therefore an ideal single parasite antigen with multiple parasite development stage target potential. TRAP is essential for the motility and invasion of hepatocytes by sporozoites (Matuschewski, 2006). These antigens have however been exploited more for the development of antibody-based vaccines that target the clinically relevant asexual blood stages. Polymorphisms in these antigens that result from sexual recombination events or point mutations however render antibody responses to one variant form of antigen less effective against other variants of the same antigen (Kusi et al., 2009; Neafsey et al., 2015; Ouattara et al., 2013).

The cellular immune response to malaria is highly dependent on the presentation of parasite-derived peptides by human leukocyte antigen (HLA) molecules to stimulate T cells. This can be exploited for vaccine design and has already been demonstrated using attenuated whole parasite vaccines that are believed to elicit significant protection-associated T cell responses (Berry et al., 2025; Epstein et al., 2017; Ishizuka et al., 2016; Jongo et al., 2021). HLA molecules are highly polymorphic, and their distribution varies markedly across different populations (Sidney et al., 2008). HLA allele diversity can therefore influence the recognition of T cell epitopes in malaria antigens, and the combined effects of malaria antigen diversity and host HLA diversity is the limited recognition of parasite antigen epitopes by the immune system, with a potential effect of reduced effectiveness of epitope-based vaccines in different populations. While the effect of parasite antigen polymorphisms can be eliminated by identifying and focusing on conserved immunogenic epitopes, the effect of host HLA diversity cannot be similarly eliminated in vaccine design (Belmonte et al., 2023; Kusi et al., 2020). Therefore, understanding the population HLA coverage of potential vaccine epitopes is crucial for designing vaccines that are effective across different populations with extensive HLA diversity.

Population coverage analysis estimates the number of people in a population who are likely to respond to specific epitopes based on their HLA types. This approach is important in multi-epitope vaccine design to identify epitopes that can be recognized by most individuals within any population. This is especially important for diseases like malaria, where the pathogen exists in many variant forms and is highly prevalent in specific regions. In our previous work, we identified several epitopes that show these unique properties by screening human PBMCs from a single locality in the southern part of Ghana with short parasite peptides from four malaria antigens (Belmonte et al., 2023; Kusi et al., 2022). In this current study, we further explore the extent of HLA recognition of sixteen of these experimentally identified epitopes, derived from CSP, AMA1 and TRAP parasite proteins, across various global populations using *in silico* predictive algorithms. We sought to identify experimentally validated CD8^+^ T cell epitopes from the CSP, AMA1 and TRAP antigens that have high HLA coverage within different populations to further affirm their potential as candidate epitopes for inclusion in universally relevant multi-epitope malaria vaccines. In addition, we also aim to identify epitopes with similar HLA coverage across different populations to inform their strategic combination in a P. *falciparum*-specific multi-epitope vaccine construct.

## Methodology

### Epitopes and HLA allele selection

Sixteen (16) experimentally confirmed *P. falciparum* epitopes derived from CSP, AMA1 and TRAP proteins and identified as promiscuous by our team were selected for global coverage analysis in this study (Table 1) (Belmonte et al., 2023). Two of the CSP peptides, CSPep4 and CSPep6, were not considered promiscuous in this study due to the one or two alleles associated with them in our analysis. These 16 epitopes were already known in literature based on epitope predictions or wet laboratory testing, and we experimentally confirmed in our previous study (Belmonte et al., 2023). All the peptides were associated with a specific group of HLA alleles based on the HLA types of the Ghanaian individuals who were recruited for our previous epitopes discovery study (Kusi et al., 2022) and on the HLA supertype classification described by Sidney et al. (2008).

**TABLE 1:** Epitopes and the selected alleles for population coverage analysis.

Epitope ID	Epitope	Selected alleles
CSPep1	ALFQEYQCY	HLA-A*74:01:01:01, HLA-A*36:01, HLA-A*30:01:01:01
CSPep2	FVEALFQEY	HLA-B*42:01:01:01, HLA-B*18:01:01, HLA-B*35:01:01, HLA-B*53:01:01:01, HLA-A*30:01:01:01, HLA-A*36:01
CSPep3	HIKEYLNKI	HLA-A*30:01:01:01, HLA-B*42:01:01:01, HLA-B*35:01:01, HLA-B*51:01:01, HLA-B*53:01:01:01
CSPep4	ILSVSSFLF	HLA-A*36:01
CSPep5	IQNSLSTEW	HLA-A*02:02:01:01, HLA-A*02:05:01:01, HLA-A*02:01:01:01, HLA-A*03:01:01:07, HLA-B*07:02:01, HLA-B*35:01:01, HLA-B*53:01:01:01, HLA-B*57:02:01, HLA-A*68:02:01:01, HLA-A*30:01:01:01, HLA-A*68:01:01:02, HLA-B*57:03:01, HLA-A*30:02:01:01, HLA-A*23:01:01:01, HLA-A*01:01:01:01, HLA-B*44:03:01, HLA-B*58:01:01, HLA-B*45:01:01, HLA-B*42:01:01:01, HLA-B*51:01:01, HLA-B*57:04:01, HLA-A*02:02:01:01, HLA-A*02:05:01:01, HLA-A*02:01:01:01, HLA-A*03:01:01:07
CSPep6	KMEKCSSVF	HLA-A*30:02:01:01, HLA-A*30:01:01:01
CSPep7	MPNDPNRNV	HLA-A*02:01:01:01, HLA-A*68:02:01:01, HLA-B*35:01:01, HLA-B*42:01:01:01
CSPep8	SANKPKDELDY	HLA-A*30:02:01:04, HLA-B*58:01:01, HLA-B*57:02:01
CSPep9	SSFLFVEAL	HLA-A*02:05:01:01, HLA-A*02:01:01:01, HLA-A*68:02:01:01, HLA-B*15:10:01
CSPep10	STEWSPCSV	HLA-A*02:01:01:01, HLA-A*01:01:01:01, HLA-A*68:02:01:01
CSPep11	VTCGNGIQVR	HLA-A*33:03:01:01, HLA-A*30:01:01:01, HLA-A*34:02:01:01, HLA-A*02:01:01:01, HLA-A*68:01:01:02, HLA-A*74:01:01:01, HLA-A*68:02:01:01
CSPep12	YANDIEKKI	HLA-A*02:01:01:01, HLA-A*68:02:01:01, HLA-B*35:01:01, HLA-B*53:01:01:01
CSPep13	YLNKIQNSL	HLA-A*02:02:01:02, HLA-A*02:01:01:01, HLA-A*68:02:01:01, HLA-B*35:01:01, HLA-B*51:01:01, HLA-B*42:01:01:01, HLA-B*53:01:01:01
AMA1ep1	TLDEMRHFYK	HLA-A*30:01:01:01, HLA-A*34:02:01:01, HLA-A*74:01:01:01, HLA-A*33:03:01:01, HLA-B*45:01:01, HLA-B*44:03:01, HLA-B*18:01:01
AMA1ep2	YLKDGGFAF	HLA-B*53:01:01:01, HLA-B*45:01:01, HLA-B*18:01:01
TRAPep1	LLACAGLAYK	HLA-A*01:01:01:01, HLA-A*03:01:01, HLA-A*30:01:01:01, HLA-A*30:02:01:01, HLA-A*68:01:01:02, HLA-B*07:02:01, HLA-B*35:01:01

### Epitope conservancy analysis

The conservancy analysis of the shortlisted experimentally validated CD8^+^ T cell epitopes (Table 1) was carried out using the “epitope conservancy analysis” tool (http://tools.iedb.org/conservancy/) of the IEDB resource. This property indicates the availability of the epitopes in a range of the sixteen (16) highly diverse strains of *P. falciparum* (PFHB3; PFML01; PFGA01; PFSN01; PFSD01; PF3D7; PFCD01; PFGB4; PFDd2; PFIT; PFKH01; PF7G8; PFTG01; PFKH02; PFGN01; PFKE01). The respective antigens with unique identifiers from the diverse strains (AMA1 (PF3D7_1133400.1; Pf7G8_110037300-t41_1; PfCD01_110038900-t41_1; PfDd2_110036700-t41_1; PfGA01_110037700-t41_1; PfGB4_110040000-t41_1; PfGN01_110038000-t41_1; PfHB3_110036900-t41_1; PfIT_110038000-t41_1; PfKE01_110038000-t41_1; PfKH01_110037800-t41_1; PfKH02_110038700-t41_1; PfML01_110038300-t41_1; PfSD01_110036100-t41_1; PfSN01_110036600-t41_1; PfTG01_110037900-t41_1), CSP (PF3D7_0304600.1; Pf7G8_030010400-t41_1; PfCD01_030010000-t41_1; PfDd2_030009600-t41_1; PfGA01_030011200-t41_1; PfGB4_030010200-t41_1; PfGN01_030010200-t41_1; PfHB3_030008400-t41_1; PfIT_030009400-t41_1; PfKE01_030009000-t41_1; PfKH01_030009500-t41_1; PfKH02_030010100-t41_1; PfML01_030009600-t41_1; PfSD01_030009700-t41_1; PfSN01_030010300-t41_1; PfTG01_030011000-t41_1) and TRAP (PF3D7_1335900.1; Pf7G8_130040300-t41_1; PfCD01_130041500-t41_1; PfDd2_130041700-t41_1; PfGA01_130042000-t41_1; PfGB4_130041800-t41_1; PfGN01_130042600-t41_1; PfHB3_130042200-t41_1; PfIT_130041200-t41_1; PfKE01_130041500-t41_1; PfKH01_130039900-t41_1; PfKH02_130038800-t41_1; PfML01_130039800-t41_1; PfSD01_130042600-t41_1; PfSN01_130038900-t41_1; PfTG01_130041600-t41_1) were retrieved from PlasmoDB (https://plasmodb.org/plasmo/app/) and submitted alongside the epitope FASTA files simultaneously for the conservancy calculation.

### Population coverage prediction

To determine the proportion of individuals in the geographic subregions; West Africa, Southwest Asia, Southeast Asia, South Asia, South America, South Africa, Northeast Asia, North America, North Africa, Europe, East Asia, East Africa, Central America, Central Africa, 78 countries and the World anticipated to exhibit immune responses to these epitopes based on the distribution of HLA types, the IEDB population coverage tool (http://tools.iedb.org/population/) was used. The IEDB population coverage tool is a valuable resource for predicting the population coverage of T-cell epitopes based on HLA allele frequency distribution in the different populations. Data on HLA binding promiscuity was extracted from our HLA allele database following genotyping of 300 study subjects from our previous study (Kusi et al., 2022). For each epitope, all the possible HLA alleles belonging to the HLA supertypes that were experimentally shown to bind to the epitope (Table 1) were selected from the HLA database for analysis. At the time of prediction, the HLA-B*15:220:01:01 allele (belonging to supertype B27) was the only allele from the classification by Sidney et al. (2008) that was not available in the IEDB population coverage database and was therefore excluded from the population coverage predictions. This exclusion affected only one peptide, CSPep9. A text file containing all sixteen epitopes and their associated HLA alleles was uploaded to the IEDB server and the populations of interest stated earlier were selected simultaneously to generate the population coverage data. Additional information on the specific population(s) for which the HLA coverage data was required was inputted and the details submitted to the IEDB server for the job execution. The output data included the percent population (HLA) coverage for each epitope as well as the number of different alleles within any population that the epitope could bind to (see Supplementary Materials 1,2 at https://doi.org/10.5281/zenodo.17210552) (Siame, 2025).

### Data analysis

Results for the selected populations from the IEDB population coverage prediction tool were organized with Microsoft Excel as proportions. Data visualization was conducted using R (version 4.3.2, R Development Core Team) to evaluate the percentage of individuals across the selected population who may potentially respond to selected epitopes. The dataset (Supplementary Materials 3,4) was imported using the read.csv function, and values representing coverage were scaled by a factor of 100 to convert proportions into percentages. Using functions from the *tidyverse* package, the data was reshaped from wide to long format with each row representing an epitope-region pair and its corresponding population coverage value. Country names were cleaned to ensure proper labeling in plots. A heatmap and global map was generated using *ggplot2*.

To investigate the similarity in population coverage profiles among epitopes, a correlation matrix was computed and visualized. The transformed population coverage dataset, with values expressed as percentages, was retained as numeric columns representing regional coverage values and epitope names were stored as row identifiers. The matrix was then transposed to allow computation of correlations between epitopes (rows). Spearman’s rank correlation method was used to capture both linear and monotonic relationships among epitopes. The resulting correlation matrix was visualized as a clustered heatmap using the *pheatmap* package, with hierarchical clustering performed on both rows and columns using Euclidean distance and complete linkage to group epitopes with similar coverage patterns. A diverging color palette (“RdBu”) was applied to reflect the direction and strength of correlations, while correlation coefficients were displayed within each tile for interpretability. In addition, statistical significance for Spearman rank correlations was determined using the t-approximation for the distribution of the correlation coefficient, as implemented in the *Hmisc* R package. All analyses were conducted utilizing additional packages including *readr, readxl, dplyr, pheatmap*, *map* and *RColorBrewer*.

## Results

### Epitope conservancy

The epitopes (Table 1) from the CSP, AMA1 and TRAP antigens of *P*. *falciparum* were assessed for their conservation across 16 highly diverse laboratory strains. The level of conservation varied among the epitopes, with CSPep1, CSPep2, CSPep4, CSPep6, CSPep9, CSPep10, CSPep11, and TRAPep1 showing 100% conservation across all strains analyzed. The lowest conservation observed was 55.56%, for peptide CSPep3 (Table 2). Detailed conservation data for each epitope within an antigen has been presented in Supplementary Materials 1 on sheets 2, 3, and 4 at https://doi.org/10.5281/zenodo.17210552.

**TABLE 2:** Conservancy of epitopes across sixteen diverse strains of *Plasmodium falciparum*.

Epitope name	Length	Number of conserved strains	Minimum identity (%)	Maximum identity (%)
CSPep1	9	16/16	100.00	100.00
CSPep2	9	16/16	100.00	100.00
CSPep3	9	2/16	55.56	100.00
CSPep4	9	16/16	100.00	100.00
CSPep5	9	13/16	77.78	100.00
CSPep6	9	16/16	100.00	100.00
CSPep7	9	15/16	88.89	100.00
CSPep8	11	6/16	81.82	100.00
CSPep9	9	16/16	100.00	100.00
CSPep10	9	16/16	100.00	100.00
CSPep11	10	16/16	100.00	100.00
CSPep12	9	4/16	88.89	100.00
CSPep13	9	1/16	66.67	100.00
AMA1ep1	10	2/16	60.00	100.00
AMA1ep2	9	4/16	88.89	100.00
TRAPep1	10	16/16	100.00	100.00

### Population coverage across geographic subregions and the world

**FIGURE 1:**
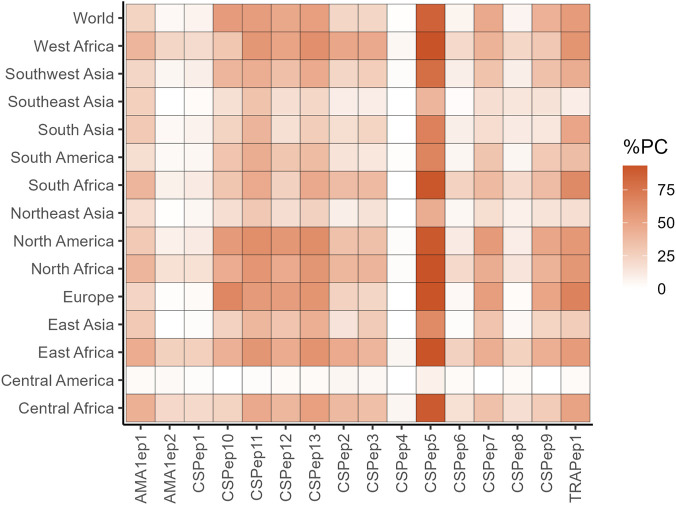
Overview of population coverage of the individual epitopes across selected geographic subregions. Data presented as a heat map with darker colour representing higher percentage population coverage. %PC; Percent population coverage.

Sixteen experimentally confirmed epitopes derived from *P*. *falciparum* CSP, AMA1 and TRAP proteins (Table 1) were analyzed *in silico* in this study. These epitopes showed HLA Class I binding promiscuity and were analyzed for HLA diversity coverage using the IEDB population coverage prediction tool. Globally, the CSP epitope CSPep5 showed the highest population coverage (>85%) followed by TRAPep1 (54.75%) and CSPep10 (54.15%). CSPep5 epitope also showed the highest percent population coverage in the European, Asian and American populations (Figure 1). Most epitopes had less than 25% global coverage, particularly in non-African populations. Generally, all epitopes were recognized by HLA types from the different global regions, except for CSPep4 which had less than 2% coverage in the Asian, European and American populations (Figure 1).

In Africa, CSPep5 had the highest coverage (89.42%–93.35%), with the highest in West Africa (93.35%) and the lowest in Central Africa (89.42%). In contrast, CSPep4 had minimal coverage (0%–5.21%) (Figure 1). CSPep13 ranked second in Central (52.39%), East (59.12%), North (57.65%), and West (61.34%) Africa, while showing 64.12% coverage in Southern Africa. TRAPep1 was the third in Central (58.42%) and West (50.22%) Africa, whereas CSPep11 ranked third in East (57.62%) and North (57.50%) Africa. In Southern Africa, CSPep13 was third (46.52%).

The observed coverage was primarily driven by alleles from A01, A02, A24, B07, B27, B44, and B58 HLA supertypes (specific alleles in Table 1). Additionally, the top five epitopes globally and in the African regions, CSPep10, CSPep5, CSPep13, CSPep11, and TRAPep1, showed high conservation (100%, 81.25%, 6.25%, 100%, and 100%, respectively) across the 16 highly diverse *P. falciparum* strains (Table 2).

### Population coverage across countries

**FIGURE 2:**
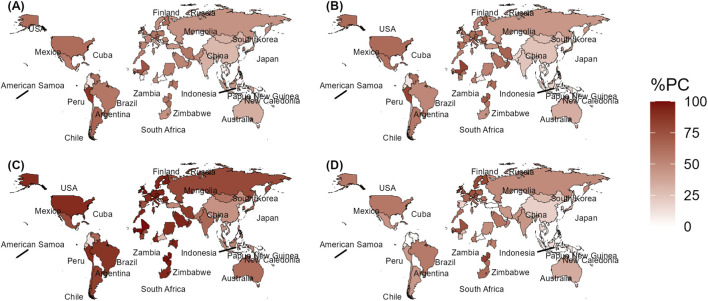
HLA coverage of CSPep11 **(A)**, CSPep13 **(B)**, CSPep5 **(C)** and TRAPep1 **(D)** across countries with available HLA data in the IEDB population coverage prediction tool. Darker colour indicates higher HLA coverage. Only countries with coverage data available are shown on the maps. %PC; Percent population coverage.

HLA coverage analysis of 16 epitopes (Table 1) across 78 countries with HLA data available in the IEDB population coverage prediction tool revealed distinct patterns of country-level recognition (see Supplementary Materials 2 at https://doi.org/10.5281/zenodo.17210552). CSPep5 consistently achieved the highest coverage in addition to CSPep13, TRAPep1, and CSPep11, whereas CSPep4, CSPep1, CSPep2, and CSPep8 were among the least recognized (Figure 2, Supplementary Materials 4,5). In Africa, where data were available for only 17 countries, CSPep5 reached 89.39% in Sudan, 92.12% in Uganda, 77.02% in Burkina Faso, and 97.24% in Zimbabwe. The least coverage for CSPep5 was 1.00% in Wales and 2.00% in the United Arab Emirates. CSPep13 showed broad coverage across 12 African countries, ranging from ∼50.00 to 80.00% in Burkina Faso, Cameroon, Cape Verde, Guinea-Bissau, Ivory Coast, Kenya, Mali, Senegal, South Africa, Tunisia, Uganda, and Zambia. TRAPep1 reached 64.12% in South Africa, 55.59% in Cameroon, 68.83% in Cape Verde, 60.46% in Morocco, 67.10% in Senegal, 56.17% in Tunisia, 82.43% in Zambia, and 62.21% in Zimbabwe, while CSPep11 covered 71.65% in Guinea-Bissau, 58.80% in Kenya, 53.93% in Sudan, 61.13% in Uganda, and 61.19% in Zimbabwe. At the country-specific level, TRAPep1, AMA1ep1, and AMA1ep2 displayed coverage between ∼10.00 and 80.00%, exceeding 50.00% in most countries except the Central African Republic, where TRAPep1 covered only 3.00%. Notably, CSPep5 maintained >50.00% coverage in low malaria risk countries including the United States, Canada, Austria, much of Europe, and Argentina, but only 27.02% in the Central African Republic, a region with relatively high malaria burden.

### Correlation clustering of closely related epitopes with respect to HLA coverage

**FIGURE 3:**
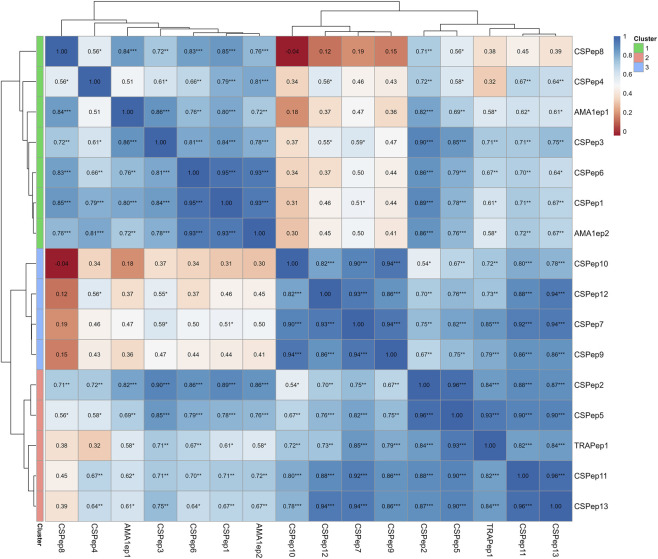
Spearman’s rank correlation of HLA population coverage. The heatmap displays the Spearman correlation coefficients (ρ) for population coverage between different epitopes. The color gradient represents the strength and direction of the correlation, with blue indicating a positive correlation and red indicating a negative correlation. Within each cell, the numerical value represents the correlation coefficient, while the asterisks indicate statistical significance levels determined by a t-test approximation: ***p < 0.001, **p < 0.01, *p < 0.05. Cells without asterisks are not statistically significant (p > 0.05). The rows and columns are clustered using Euclidean distance and the complete linkage method to group epitopes with similar coverage patterns.

To identify epitopes with similar HLA coverage that are significant in relation to the different geographic regions, Spearman’s rank correlation and t-approximation for the distribution of the correlation coefficient were performed (Figure 3). Several significant correlations and three distinct clusters of epitopes with similar coverage were identified. Cluster one, which contained five epitopes (CSPep1, CSPep3, CSPep4, CSPep6, CSPep8) had the least HLA coverage while cluster two contained five epitopes (CSPep2, CSPep5, CSPep11, CSPep13 and TRAPep1)and had the highest HLA coverage Cluster three contained four epitopes (CSPep7, CSPep9, CSPep10 and CSPep12) and had moderate HLA coverage.

## Discussion

Currently licensed malaria vaccines RTS,S and R21 target the pre-erythrocytic stage of the parasite and reduce the parasite load that reaches the blood stages to cause the clinical symptoms of the disease. This is an essential strategy for reducing the burden of disease, and the two vaccines are described as anti-disease vaccines (Laurens, 2019; Ngou et al., 2024; Oduoye et al., 2024). Protection offered by both vaccines is however mediated mostly by antibodies (Datoo et al., 2022; Kurtovic et al., 2024; 2020), with very limited CD8^+^ T cell involvement. Maintaining these antibody responses will require a strong T cell response. Furthermore, a vaccine that can target the parasite within hepatocytes will be desirable as it will also halt parasites growth before it reaches the symptomatic blood stages. Whole sporozoite vaccines have demonstrated capacity to achieve this through the induction of potent T cell responses against intracellular parasites (Berry et al., 2025; Ishizuka et al., 2016; Jongo et al., 2021). Identifying the targets of these T cell responses will therefore be essential for developing relatively simpler and cost-effective epitope-based vaccines with the potential of protecting against most variants of *P. falciparum*. The development of an epitope-based pan-vaccine against *P. falciparum* therefore requires careful consideration of both host HLA diversity and parasite epitope diversity.

When multi-epitope vaccines are rationally designed, they could provide broad immunity as they will be designed to activate both B- and T-cells. In addition, wide population HLA coverage can enhance targeting multiple stages of the *P. falciparum* parasite with greater efficacy, increased duration of protection and limit immune evasion (Beutler et al., 2022; Sun et al., 2025). However, the parasite antigenic variation, complex lifecycle and the potential of antigen immunodominance, where the immune system is focused on only a few dominant epitopes, could limit the success of *P. falciparum* multi-epitope vaccine design. Careful engineering design is needed to overcome these potential challenges that may arise (Chick et al., 2025).

In this study, we performed *in silico* HLA recognition analyses of experimentally identified unique epitopes from three *P*. *falciparum* antigens by assessing their global and country-specific HLA coverage using tools from the IEDB platform. This was to confirm their relevance for inclusion in a universal multi-epitope vaccine. This study revealed significant geographic variation in population HLA coverage of our selected malaria epitopes, reflecting the polymorphic nature of HLA molecules and their differential distribution among human populations (Sidney et al., 2008). Our analysis identified three epitopes with particularly promising characteristics: IQNSLSTEW (CSPep5), LLACAGLAYK (TRAPep1), and STEWSPCSV (CSPep10), which demonstrated superior population HLA coverage at the geographic subregion level (>54%) and in African regions (>50%). At the country-specific level, CSPep5, CSPep13, TRAPep1 and CSPep11 had relatively higher HLA coverage among the selected epitopes. The consistently higher coverage by CSPep5 reaffirms its potential as a CD8^+^ stimulatory epitope for inclusion in an epitope-based vaccine construct, whereas the increased coverage by TRAPep1, AMA1ep1, AMA1ep2 and low coverage by CSPep5 in the Central African Republic, Wales and the United Arab Emirates populations affirm the diversity of HLA molecules across populations. CSPep5 high coverage in high-risk malaria countries including Sudan, Uganda, Burkina Faso, and Zimbabwe, underscore its inclusion in region-specific multi-epitope vaccine formulations. However, the lack of HLA data for high-risk countries such as Nigeria and Ghana in the IEDB could limit the justification for inclusion of potential epitopes into vaccine construct. This serves as an opportunity for these high-risk countries to invest in HLA typing of their population and making the data available in databases such as IEDB to support rational epitope-based vaccine design.

All 13 CSP epitopes analyzed in this study are found in the CSP fragments used for the construction of the RTS,S and R21 vaccines and are outside of the NANP repeat region of CSP. Of these, CSPep3, CSPep5, CSPep6, CSPe7, CSPep8, CSPep10, CSPep11, CSPep12 and CSPep3 are fully conserved, while CSPep1, CSPep2, CSPep4 and CSPep9 epitopes have amino acid variability (Supplementary Materials 1 (Siame, 2025)). Epitopes outside of the NANP repeat regions have been reported to play a role in activating CD8^+^ T cells following vaccination with the RTS,S and R21 vaccines (Collins et al., 2017; Epstein et al., 2011). These findings suggest that the conserved epitopes identified in our study, along with selected epitopes from TRAP and AMA1, may contribute to a stronger and more durable CD8^+^ T cell response against *P. falciparum* when included in a multi-epitope vaccine. These epitopes could be prime candidates for inclusion in a universal multi-epitope vaccine formulation due to their combined desirable properties of broad HLA recognition and amino acid conservation across multiple *P. falciparum* variants (Table 2). We however acknowledge that our current estimation of conservancy in the epitopes we have assessed in this study may be overstated, as conservancy assessment of the epitopes was performed a while ago, with only sixteen highly diverse laboratory variants.

The CSP-derived epitope CSPep5 demonstrated exceptional population coverage (>85% globally and up to 93.35% in West Africa), attributable to its broad binding affinity across multiple HLA supertypes including A01, A02, A24, B07, B27, B44, and B58 (Belmonte et al., 2023). In striking contrast, epitopes such as CSPep4 showed minimal coverage (<5% in Africa and <2% elsewhere), illustrating how restricted HLA binding profiles can substantially limit immune recognition (Heide et al., 2019). Most of the HLA alleles used in our HLA coverage analysis belong to common supertypes previously identified in our participants (Belmonte et al., 2023; Kusi et al., 2022).

While high population HLA coverage represents a crucial criterion for vaccine candidate selection, epitope conservation across diverse parasite strains is an equally critical factor. Notably, certain epitopes with moderate population coverage, such as CSPep13, demonstrated complete conservation (100%) despite being presented by a limited set of HLA alleles (Belmonte et al., 2023). Although the epitope demonstrates limited HLA promiscuity relative to others, the amino acid conservation (Table 2) could justify their potential inclusion in vaccine constructs after functional trial assays are conducted as they may provide broad protection against diverse *P. falciparum* strains. A strategic combination of both high HLA coverage and epitope conservancy in multi-epitope vaccines therefore could represent a balanced approach to universal vaccine construction, ensuring broad immune recognition while simultaneously mitigating the risk of parasite strain-specific immune evasion. This combination would address two critical challenges in this vaccine design approach: (1) the need for broad population coverage across diverse HLA backgrounds, and (2) protection against antigenic variation in circulating parasite strains. Notably, all three epitopes derive from either CSP or TRAP–antigens expressed during the pre-erythrocytic stage suggests they could be particularly effective in preventing initial infection.

The findings also underscore the importance of considering non-endemic populations in vaccine development strategies. Although severe malaria transmission remains concentrated in sub-Saharan Africa, individuals from regions with low or no endemicity such as Europe and the Americas require effective vaccination when traveling to these high-risk areas. The pronounced disparities in epitope binding coverage between African and non-African populations highlight the necessity for inclusive vaccine formulations that account for global HLA diversity. For instance, the AMA1-derived epitope AMA1ep2 showed moderately higher coverage in West, East and Central Africa (greater than 20%), but lower coverage (5% or less) in other regions except North America (8.46% coverage), demonstrating the challenges inherent in achieving universal protection without carefully tailored epitope selection for vaccine design (Zhou et al., 2024).

While epitopes with broad HLA binding profiles such as CSPep5 represent promising vaccine candidates, conserved epitopes with more limited coverage should not be overlooked, as they may contribute significantly to long-term protection against evolving parasite strains. Ultimately, identifying additional epitopes with these unique characteristics relating to HLA promiscuity binding and amino acid conservation across diverse parasite strains will be essential for designing universally applicable malaria vaccines. This strategy would leverage the strengths of each epitope while mitigating their individual limitations. Immunogenicity assessment of vaccines designed on this basis are currently ongoing in animal models, with potential to proceed to human trials in the future. By adopting a globally inclusive strategy that addresses the needs of both endemic and non-endemic populations, these next-generation malaria vaccines can make substantial progress toward achieving equitable and durable protection against *P. falciparum* infection worldwide. The scope of such approaches can also be widened to search for epitopes that may be conserved across multiple *Plasmodium* species for the development of pan vaccines against multiple malaria parasite species.

An important limitation of our study is the use of only lab-adapted parasite strain sequences in the conservancy analysis. Although these labs strains were selected to represent, to our knowledge, the greatest extent of diversity in P falciparum parasites, an expansion of this to include filed isolate sequences may have added to the representativeness of the data. Additionally, the unavailability of HLA sequence data from majority of African countries in the IEDB database limits the generalizability of our findings from this study. This is especially important because of the greater HLA diversity in Africa. These notwithstanding, the data as presented has relevance for the design of future universal vaccine candidates against malaria. Furthermore, the approach can be adapted for analysis and identification of broad coverage epitopes for other diseases where antigenic polymorphism have limited the progress of effective vaccine design.

## Data Availability

The datasets presented in this study can be found in online repositories. The names of the repository/repositories and accession number(s) can be found in the article/Supplementary Material.
