# Supplementary figures and images for: Population HLA coverage of experimentally characterized P. falciparum CD8 + T cell epitopes as potential components of a multi-epitope malaria vaccine construct: an in silico insight
Source: Front Bioinform. 2026 Mar 12;6:1722563. doi: 10.3389/fbinf.2026.1722563 (PMC13018126; doi:10.3389/fbinf.2026.1722563)

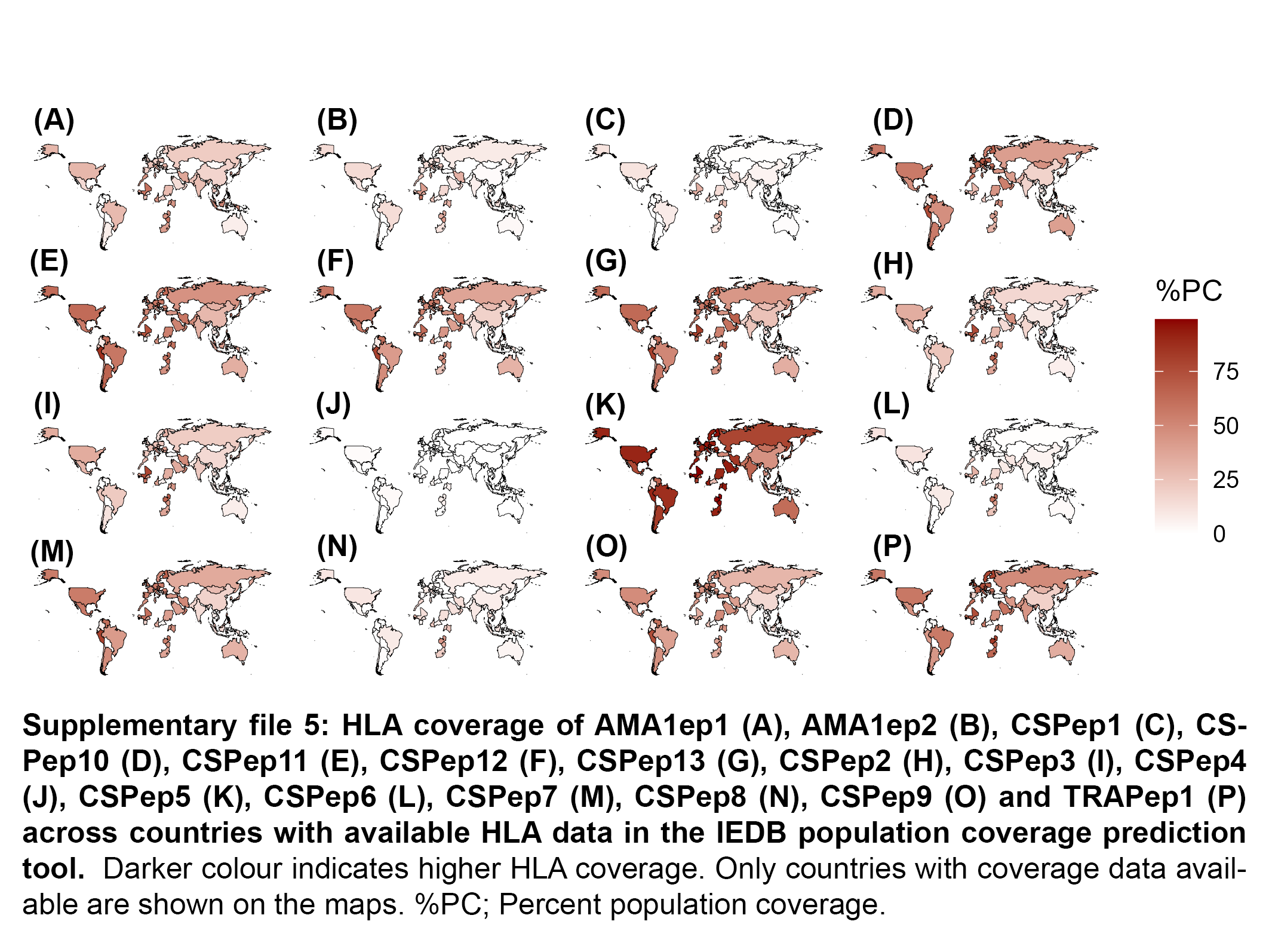

Supplement: Supplementary file 3 [file Image1.png]
